# Supplementary material for: Molecular characterization of nervous system organization in the hemichordate acorn worm Saccoglossus kowalevskii
Source: PLoS Biol. 2023 Sep 19;21(9):e3002242. doi: 10.1371/journal.pbio.3002242 (PMC10508912; doi:10.1371/journal.pbio.3002242)
Supplement: Attachment — Submitted filename: Response to Reviewers.docx [file pbio.3002242.s005.docx]

**Response to reviewers.**

We first would like to really thank the reviewers for the constructive comments on the written document and the work that was involved in helping us craft a better manuscript. In some cases, these comments were very detailed and incredibly helpful to help make the manuscript more readable and more accessible to a general audience. Below we outline the detailed response to comments/suggestions, point by point.

**Reviewer #1:**
This paper provides a detailed description of the anatomy of the nervous system in juveniles and adults of the hemichordate Saccoglossus kowalevskii nervous system using in situ hybridization, immunostaining and transgenic reporters for multiple neuronal markers (including neurotransmitters and neuropeptides). Although various aspects of hemichordate NS organization have been previously described, the present study provides the most comprehensive overview of the hemichordate NS to date and reveals new insights into its organization, for example by highlighting several unexpectedly long range connections between cell populations. The paper will therefore present a valuable resource for anyone interested in the evolution of nervous systems.

In its current stage, however, the paper suffers from poor labelling of the figures and unclear or ambiguous anatomical descriptions or figure annotations, which makes it difficult for the uninitiated reader to follow the descriptions. This necessitates a thorough revision of the text and figures as suggested in my detailed comments below.

Detailed comments:
- 57: delete "now"

Corrected in text

- 79/80: one ventral cord and one dorsal cord

Corrected in text

- Fig.1:
*       label sections in A with panel numbers;

Transverse sections are now labeled in Figure 1A.

*       explain all abbreviations (e.g. PDC, PB, PS);

Abbreviations are now included in the figure legend.

*       the placement of labels often is ambiguous - use arrows or lines to disambiguate eg it is not clear which structure is ACR)

lines added to more clearly annotate the figure.

*       always indicate orientation of sections (eg. where I dorsal in B, C?)

We standardized orientation with dorsal aligned to the top of the panel and more clearly outlined in the figure legend.

*       I'm confused about the staining domains in D: there are two domains in the ectoderm on top, one at the bottom, what are they?

They are the anterior collar ring that is caught at multiple places in section due to the curvature of the collar. They are now labelled as ACR.

*       K-M; label structures in sagittal sections:

Sagittal sections are now labeled in Figure 1A.

*       N,O: label proboscis, collar and collar cord

Labels are now included in figure.

- 129: "expression of GRN" is awkward wording

We added detail to clarify that we are using GRN to mean a group of genes that specify neuronal cell types in the nervous system, and how this gene regulatory network patterns the CNS.

- 130-132: almost nothing is known about regulatory links in GRN of Saccoglossus

We agree to some extent with this issue; however, functional studies have resulted in major transformations in body plan organization that includes the nervous system. (Pani et al. 2012, Darras et al. 2018 and Lowe et al. 2006). So, while there has not been a set of functional studies directly testing effects on nervous system, the loss of body regions during Wnt manipulation for example, clearly also affects the nervous system. However, we clarify in the text that more links to nervous system from functional studies are needed.

“Functional studies have demonstrated the key role of these regulatory networks in body plan patterning (Darras et al 2018, and Pani et al 2012, Lowe et al 2006) including nervous system regionalization, yet there has been no explicit test of how this network regulates nervous system specifically.”

- 196: where are elav cells in gut? Label in Fig. 1!

We labeled the pharyngeal epithelium showing the staining in Fig 1M.

- 215: It needs to be stated here that all the following data are based on in situ hybridization and table Suppl. 1 needs to be cited. The wording in this section often suggests misleadingly that you describe the distribution of neuropeptides, where you really describe the expression of genes encoding their unprocessed precursors. Explain whether each of these genes encodes only a single neuropeptide. Please also explain why some of the staining in Fig 2-3 appears as NBT/BCIP signal, while other staining appears fluorescent (HCR?)

We have amended the wording in the text referring to neuropeptide localization, as suggested, to be clear about what the localizations represent. However, in line 222 we already state that the data in this section are based on *in situ* hybridization. Each of the propeptide sequences are from different ESTs and represent individual transcripts.

- Fig.2:
*       You need to clarify that these are in situs;

This explanation is now added in the figure legend.

*       explain briefly which panels are based on HCR or on NBT/BCIP;

This explanation is now added in the figure legend.

*       explain whether blue counterstain is DAPI;

This explanation is now added in the figure legend.

*       all abbreviations must be explained

Abbreviations are now included in the figure legend.

*       C'': many TH cells do not coexpress DAT

We explained in the text that the TH+ cells that do not co-express DAPT are probably other catecholaminergic neurons.

*       C''': TH and DAT staining barely visible

We added a higher magnification of the base of proboscis and anterior collar to improve localization of DAT+ cells in the proboscis base.

*       What are open arrows in F/F'?

All closed arrows point to expression in the proboscis base and open arrows to the anterior collar. This was clarified in the figure legend. Same for Figure 3.

*       What are we supposed to see in H?
We increased the magnification so the scattered cell bodies in the ectoderm can be seen clearly and the narrative in the figure legend clarified.

- 244: "TH cells at the base of the proboscis". It is not clear which cell group in Fig. 2 you mean; no reference to Fig. panel

Reference to Fig 2C’’ was added for clarity.

- 300: "dense anterior expression": this is not visible in Fig. 2H

We increased the magnification so the cell bodies can clearly be seen.

- 336: delete "P"

This typo was deleted.

- Fig. 3:
*       Again, emphasize that we see in situs here (and which kind of staining)

This was added in the figure legend for clarity in both Figure 2 and Figure 3.

*       The legend is written in a confusing way; please follow the following template: "(X, X'): Expression of … in early (X) and late (X') juvenile"

This formatting suggestion was addressed in Figure 3 legend.

*       Inset in G' is smaller than main panel but shows the same view, so please remove

Inset was removed.

*       I': how can you see the ventral cord in a dorsal view?

The main figure in I’ is a lateral view and the inset panel a dorsal view. This was clarified in the figure legend.

*       What do the arrowheads in J, K, Q mean?

White-filled arrows point to the proboscis base in all imaged in Figure 3 and the unfilled arrows point to the anterior collar. This was clarified in the figure legend.

*       N': if inset shows "RNA-based probe" what is shown in main panel?

the description was changed to “chromogenic” to distinguish between this and HCR. This was added in the figure legend for clarity in both Figure 2 and Figure 3.

*       N-O: location/orientation of insets unclear

Text was added in the figure legend for clarity.

*       L: Corticotropin (not -trophin)

Typo was corrected in text

*       R-R'': not clear , which, if any,  cells are double labeled; show individual channels

Fig 3R now show all merged channels (S) along with individual channels (S’-S’’’).

- 365: why is vasotocin not shown next to orexin?; they are discussed together in text

they should be in the order they are discussed.

Figure was reorder to coincide with the main text in the results.

- 376: gill slits and ciliary band should be labeled in Fig. 3 J'

These annotations were added to Figure 3 J’

- 409: posterior projections not clearly visible in Fig. 3 N

We added white arrows in the inset panel to point out the visible neurite projection from these cells.

- 441: with a monoclonal

Typo was corrected

- Fig. 4:
*       Again it is not clear what we see here; if all of these panels are based on fluorescent immunostaining, why is the signal black in A and white in B etc?

We used inverse color image on some panels because it showed better expression detail. This was not done when we used DAPI as a counterstain as in B.

*       Label anterior collar ring and the multiple structures visible in collar in A - label the ACR and posterior collar ring.

Serotonin doesn't light up these structures

*       For clarity, I would suggest to discuss this overall organization of the nerve tracts earlier in the manuscript, following the description of elav.

We prefer the current organization where the distribution of cell bodies and their organization is outlined first, and projections are considered separately.

*       Is the description for D and E switched? Where is the dorsal cord? What are the circumferential fibers in D?

Apologies, Yes, D and E were accidentally switched - it has been corrected.

*       F: if synaptotagmin antibody labels neurites, why can we not see individual neurites here?

Neurites are bundled in most of the plexus making it difficult to track individual neurites. The transgenes in later figures are incorporated mosaically and DiI injections are taken up selectively allowing for differentiation a

*       J,K,L,M: orientation unclear

The DiI injections were directed in the mid proboscis region. The AP axis is indicted in J-K, but is uncertain in L-M

*       J,K: what do the arrows mean?

Arrows have been removed and the legend more thoroughly describes that neurite projects show structure along the apicobasal axis of the plexus.

- 476: I cannot see the "regularly spaced connectors" -

they are only visible in E, not D. We corrected the text.

- 478-483: I cannot follow the description here. The circumferential neurites in Fig. 4 D are not described. I don't understand what is meant by "series of layered, interconnected tracks"

We edited the text for clarity.

“ a complex architecture of thicker neurite bundles forming a more disordered mesh that shows clear organizational differences along the basal/apical axis of the plexus”

- I could not run the supplementary movies, so cannot comment on their quality

Movies have been resubmitted.

- "The results from 1E11": awkward wording

This was corrected in the text.

- 495: please discuss why there may be "little resolution of individual neurites"

Corrected in text. The tight neurite bundles make it difficult to trace individual neurites.

- Fig. 5:
*       Label gill slits in A

Labels added to figure

*       B: from which region is this?

Orientation was added in figure. This is a 3GS juvenile showing neurites projecting into the neural plexus, just posterior to the collar.

*       D: why is anterior ring not included here?

Anterior ring was added in figure.

*       I,J Orientation unclear; is the proboscis removed in I?

annotations added on figure and yes, the proboscis is removed in I

*       K this shows transverse and not sagittal sections

This was corrected in the text

- 557/558: distribution of

Corrected in the text

- 583: indicate location of endodermal plexus in the figure

Endoderm plexus label was added in Fig 5O.

- 600: Fig 6Aiii (not 56iii)

Typo corrected

- Fig.6
*       In previous figures, panels showing details of A have either been labelled B,C… or A', A''; here now they are labelled Ai, Aii… - please be consistent
This format is now changed to be more consistent with the other figures.

*       A': please label gill slits

Labels added in figure

*       B-E, G: orientation unclear

Anterior to posterior orientation is from left to right, this was noted in the figure legend.

- 617: anterior ring not visible/labelled in Fig. 6C

Label added

- 663/664: please add parentheses and Fig. number to "white arrows in inset" and "grey arrows in inset"

Corrected in text

- 672: repeats previous statement in 666

Sentence in line 666 was removed to prevent repetition.

- Fig. 7:
*       Explain why signal black in some panels, white in others

Expression is easier to detect in some figures with a color inversion. This is explained in the figure legend.

*       Is blue staining DAPI?

Yes, this is described in the figure legend.

*       There are no re arrows only white and grey and they need to be explained

This was an error. There are white and gray arrows, and the text was corrected to reflect this.

*       Box for I in panel A is missing

Box I is present, no change was made.

*       D: Spelling "Pseudounipolar"

Typo was corrected.

- Fig.8:
*       Labeling of panels (A,B,C…) is missing
*       Arrowheads not explained

Figure 8 labels and legend were corrected.

- Fig. 9: Is blue staining DAPI?

This detail was added in Figure 7 and Figure 9 legend.

- 773: replace "with " with "were"

Corrected in text

- 795: reword sentence "epithelium of … neurotransmitters…"

Corrected in text

- 802: delete "plan"

Corrected in text

- 825: delete "and"

Corrected in text

- 840-856: The discussion here makes no sense to me. In vertebrates the posterior pituitary forms as an extension from the hypothalamus but the anterior pituitary from the adenohypophyseal placode, while the hypothalamic GnRH neurons derive from the olfactory placode. Which regions of the hemichordate NS are here proposed to be homologous to what? Please note also that TH cannot be considered a specific hypothalamic marker (and I'm not sure about calcitonin).

We are raising the possibility of a region at the base of the proboscis sharing some functional similarities with the vertebrate hypothalamus/pituitary. We cannot make detailed comparisons to the specific vertebrate regions due to the preliminary nature of the data and a lack of specific pituitary neural markers. If this region functions as a neurosecretory center, then we need antibody data for the neuropeptides to investigate projections and some EM data. We are actively investigating this, but prefer to keep this comparison speculative. There are rarely unique markers of any neural structure, but the clustered expression of Orexin, CRH, TRH, Calcitonin and GnRH together in the same region justifies raising this comparison.

- 910: cholinergic neurons were not described here. Is it possible that motor neurons, which innervate the muscles and possibly penetrate the basement membrane are cholinergic?

Although this is a good suggestion and a possibility, that we did not see any neurites penetrate the basement membrane from the synapsin:eGFP transgene, suggests that any cholinergic neurons that were randomly labeled with this transgene do not cross this barrier. However, future studies should generate a cholinergic transgene to test this. This rationale was added in the text.

- 1020: what were the NBP/BCIP concentrations?
These ratios are already in the text. We used 1.6uL BCIP/2.7uL NBP.

**Reviewer #2:**
In this study, the authors present the first molecular characterization of the Saccoglossus kowalevskii nervous system, using a combination of RNA in situ hybridizations, transient transfections with fluorescent reporters, and immunostainings. The images are wonderfully detailed and the text is well-written and thorough. It is clear that this paper is already highly polished. This represents an important characterization of a nervous system in a key branch of deuterostome phylogeny. It is therefore of great interest to a broad readership. I truly do not have any substantial criticisms or comments to be addressed. I fully recommend its publication in PLoS Biology.
We thank Reviewer #2 for their time support of this manuscript.

**Reviewer #3:**
[IMPORTANT: This reviewer has attached an annotated version of your PDF]

The manuscript by Andrade Lopéz et al. provides a detail atlas of the nervous system of a hemichordate, a deuterostome animal of key relevance in understanding the evolution of the chordate nervous systems. This highly detailed anatomical of high quality study provides new insights into the organisation of the nervous system of these organisms. The authors carefully describe their findings and provide a balanced discussion of their relevance. The paper will be of interest to those interested in evo-devo, emerging marine animal models and the evolution of nervous systems.

The study uses a pan-neural marker and several neuron-type specific markers labeling various neurotransmitter or neuroendocrine pathways and specific proneuropeptides in juvenile and adult specimens. The authors use whole-mount stainings, tissue sections and tissue 'peals' to provide whole-body overviews and more detailed regional views at cellular resolution of nervous system organisation. This manuscript is the to date most comprehensive compendium of molecular nervous system characterization in this enigmatic animal group. The authors combine immunohistochemistry, whole mount in situ hybridization, multi-color HCR, dye-filling and transient transgenesis.

The authors also extensively reviewed the available data about overall nervous system architecture in hemichordates and placed their findings in a broad context.

The manuscript is of high quality and will provide a valuable resource for researchers working on nervous system evolution and hemichordate biology.

Minor comments:

The text is very long and the authors may want to consider shortening it or reorganising it. Some sections that would belong to the introduction or discussion are in the main text etc. See pdf for more detailed comments.

Figure 3 is very dense, the authors could consider breaking it up into two figures.

We prefer to keep the figure as a single figure. Yes, it is data rich, but we don’t see how splitting into two figures helps clarify the details, and the text really justifies keeping the data together in a single figure.

In the pdf Figure 8 seems to have lost most of its labels, please check
This was an uploading error that will be corrected in the final submission

In Figure 8 the figure legend is incomplete, lacking the description of the lower two panel-rows
Complete figure labels and text were added, as suggested.

Please see the attached pdf for more detailed comments on the manuscript
All of reviewer #3’s suggestions were addressed in the Revised Article with Track Changes document.

"Our data provides some insights into this question, but also raises many others." - many questions

**Reviewer #4:**

This is an excellent report that warrants publication in a leading journal. The field of "evo-neuro" has long been in need of a comprehensive analysis of how a "neural net" nervous system could be organized, and this submission answers this need in spades. In addition, the manuscript provides a road map to critical future studies, such as investigating the possibility of a HPA-like neurosecretory system and the neurophysiological status of nerve control over muscle contraction. Finally, as one might imagine given the deep scholarship of the submission, it is appropriately nuanced in addressing the issue of nervous system centralization in these worms.

Minor points: To make the Figures readable for casual inspection, the authors should italicize gene names in the panels. Some author lists appear to be incomplete [#46, #50].

References were corrected for format and typo errors
